# Control of sulfur partitioning between primary and secondary metabolism in *Arabidopsis*

**DOI:** 10.3389/fpls.2012.00163

**Published:** 2012-07-19

**Authors:** Stanislav Kopriva, Sarah G. Mugford, Patrycja Baraniecka, Bok-Rye Lee, Colette A. Matthewman, Anna Koprivova

**Affiliations:** Department of Metabolic Biology, John Innes Centre, Norwich Research Park,Norwich, UK

**Keywords:** sulfate assimilation, cysteine, glucosinolates, adenosine 5′-phosphosulfate, sulfotransferase, sulfated metabolites

## Abstract

Sulfur is an essential nutrient for all organisms. Plants are able to take up inorganic sulfate and assimilate it into a range of bio-organic molecules either after reduction to sulfide or activation to 3′-phosphoadenosine 5′-phosphosulfate. While the regulation of the reductive part of sulfate assimilation and the synthesis of cysteine has been studied extensively in the past three decades, much less attention has been paid to the control of synthesis of sulfated compounds. Only recently the genes and enzymes activating sulfate and transferring it onto suitable acceptors have been investigated in detail with emphasis on understanding the diversity of the sulfotransferase gene family and the control of partitioning of sulfur between the two branches of sulfate assimilation. Here, the recent progress in our understanding of these processes will be summarized.

## INTRODUCTION

Sulfur is essential for life as a component of proteins in the amino acids cysteine and methionine, a large number of co-enzymes and prosthetic groups as well as in many natural products of the secondary metabolism (Takahashi et al., 2011). The particular characteristic of sulfur, leading to its frequent occurrence in various compounds, is its ability to readily change its oxidation state. In nature, the major form of sulfur is the oxidized inorganic sulfate, however, most of the bio-organic compounds of primary metabolism contain the reduced form of sulfur as organic sulfide or thiol. Thus, the sulfate entering living organisms has to be assimilated, i.e., reduced and incorporated into organic matter. Not all organisms are able to cover their needs by assimilating sulfate, most notably all metazoans and most microorganisms adopting a parasitic lifestyle, in which sulfate reduction seems to be one of the first pathways being lost (Patron et al., 2008). Thus, plants (and algae) together with fungi and bacteria, which are capable of sulfate assimilation, play a crucial role in the food chain and in the biogeochemical cycle of sulfur.

**FIGURE 1:**
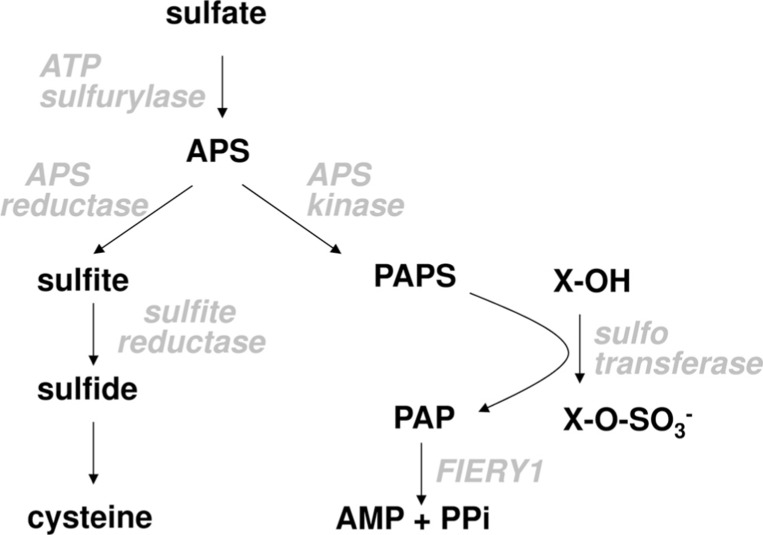
**Scheme of plant sulfate metabolism**.

Plant sulfur metabolism starts with taking up inorganic sulfate (**Figure 1**). The uptake is facilitated by sulfate transporters present in plasma membranes. Different cells possess different complements of individual sulfate transporters depending on the tissue and developmental stage (Buchner et al., 2004; Takahashi et al., 2011). Sulfate entering root cells can be rapidly moved through the cortex into the xylem and transported into the above ground plant organs or it can be directly utilized in the roots. Inside the cell it can be transported into the vacuole for storage or used directly for assimilation. Because sulfate is very stable, before assimilation it has to be activated. This is achieved in a reaction with ATP sulfurylase, in which sulfate replaces pyrophosphate in the ATP molecule. The resulting adenosine 5′-phosphosulfate (APS) is a branching point in primary and secondary sulfate assimilation. APS can either be reduced by APS reductase to sulfite in the primary sulfate assimilation pathway, or it can be phosphorylated by APS kinase to 3′-phosphoadenosine 5′-phosphosulfate (PAPS). PAPS is the active sulfate donor for the incorporation of sulfur into a variety of secondary products. Sulfite is reduced by sulfite reductase to sulfide, which is the form of reduced sulfur incorporated into the amino acid skeleton of *O*-acetylserine to form cysteine, the first product of primary sulfate assimilation (**Figure 1**). Cysteine can be used for protein and peptide synthesis or as a reduced sulfur donor for biosynthesis of methionine and a large range of co-enzymes and co-factors.

Sulfate assimilation is an essential process in plants: reduced expression of genes for several steps of the pathway lead to strong growth phenotypes, e.g., sulfite reductase (Khan et al., 2010), APS kinase (Mugford et al., 2009), or serine acetyltransferase (Haas et al., 2008), complete knock-outs are lethal (Watanabe et al., 2008; Mugford et al., 2010). The pathway is strongly regulated according to the demand for reduced sulfur and availability of various sulfur sources (reviewed in Takahashi et al., 2011). The regulation of the reductive part of sulfate assimilation has been extensively studied in the last two decades leading to a very good understanding of the responses of individual genes and enzymes to various environmental conditions and changes in metabolite levels. The availability of genetic resources in *Arabidopsis* allowed more precise defining of functions of individual members of gene families encoding various steps of sulfate assimilation to cysteine (reviewed in Kopriva et al., 2009). On the other hand, much less attention was paid to the PAPS branch of sulfate assimilation and synthesis of sulfated compounds. The partitioning of sulfur into the primary (reductive) and secondary (sulfated) assimilation represents an important step controlling the availability of this nutrient for synthesis of numerous compounds and has been addressed only very recently (Mugford et al., 2009, 2011). Here, we will summarize and discuss new findings concerning the secondary branch of sulfate assimilation, particularly the role of APS kinase and APS reductase in control of sulfur flux through the two branches of sulfate assimilation and a possible mechanism of such control.

## SULFATED COMPOUNDS AND SULFOTRANSFERASES

Whereas sulfur in primary metabolites, such as cysteine, methionine, glutathione, and most co-enzymes, is in its reduced form, plants synthesize a number of secondary metabolites incorporating oxidized sulfur. Such sulfated compounds form a diverse group of secondary metabolites important for crop fitness and stress defense and also for human diet and health. The increasing, but still limited number of plant sulfated compounds with known function is in sharp contrast to the importance of those identified to date. The transfer of the functional sulfo group to hydroxylated substrates, i.e., sulfation, is catalyzed by sulfotransferases (SOT). In mammals, sulfation is a major contributor to the homeostasis and regulation of numerous biologically potent endogenous chemicals, such as catecholamines, steroids, and iodothyronines, as well as to the detoxification of xenobiotics (Coughtrie et al., 1998). In bacteria, sulfation is essential for the signaling of rhizobial nod factors to the plant (Truchet et al., 1991). In plants, a large proportion of the known sulfated metabolites play various roles in plant defense against biotic and abiotic stress. A well-studied example of such compounds is the glucosinolates, which participate in defense against herbivores and pathogens in Brassicales (Halkier and Gershenzon, 2006). They are responsible for taste and flavor of cruciferous vegetables and possess an anti-cancer activity (Fahey et al., 2001; Mithen et al., 2003). Sulfation is the last step in synthesis of the glucosinolate core and is essential for their biological activity, as it enables formation of the reactive volatile products upon reaction with myrosinase. Another large group of medically important sulfated compounds are sulfated flavonoids, present in more than 250 species of 32 families (Barron et al., 1988), where they are involved in detoxification of reactive oxygen species and regulation of plant growth (Varin et al., 1997). Several other sulfated compounds were shown to directly participate in plant defense against pathogens: a sulfated derivative of jasmonic acid identified in *Arabidopsis* (Gidda et al., 2003) or sulfated β-1,3-glucan oligosaccharides (Ménard et al., 2004) that induce salicylic acid defense signaling. Small sulfated peptides, such as phytosulfokines, PSY1, and RGF are important regulators of plant growth (Matsubayashi and Sakagami, 1996; Amano et al., 2007; Matsuzaki et al., 2010).

The SOT-catalyzed sulfation requires PAPS as the sulfate donor and a compound with a free hydroxyl group as an acceptor. Multiple SOT isoforms are found in higher Eukaryotes because of the structural diversity of the biological acceptors of the sulfate group. The SOT family in *Arabidopsis* consists of 18 members divided into seven groups according to sequence similarity (Klein and Papenbrock, 2004). Only about half of these isoforms have been assigned a substrate specificity and/or physiological function. The AtSOT16, AtSOT17, and AtSOT18 isoforms are responsible for sulfation of desulfo-glucosinolates (Piotrowski et al., 2004; Hirai et al., 2005) with a broad substrate specificity, but clear prefernce of AtSOT16 for aromatic precursors (Klein et al., 2006). The AtSOT15 was shown to catalyze the sulfation of 11- and 12-hydroxyjasmonate, but the closely related (based on sequence identity) AtSOT14 was inactive with this substrate (Gidda et al., 2003). AtSOT10 and AtSOT12 are involved in sulfation of brassinosteroids (Marsolais et al., 2007) and/or salicylate (Baek et al., 2010), while the preferred substrates of AtSOT5 are flavonols (Gidda and Varin, 2006). The remaining SOTs are of unknown function. However, all evidence on SOT substrate specificity was obtained *in vitro* in studies with recombinant proteins, and may not reflect the situation *in vivo*. Similarly, not much is known about regulation of SOTs, except that in agreement with the potential role of sulfated compounds in plant defense, the mRNA levels of *AtSOT12*, *AtSOT15*, *AtSOT16*, and *AtSOT17* significantly increased upon treatment with jasmonate (Gidda et al., 2003). *AtSOT12* mRNA was induced also by salicylic acid and by interaction with bacterial pathogens and elicitors, whereas *AtSOT16* mRNA level responded to coronatine, an analog of octadecanoid signaling molecules, and to ACC, the precursor of ethylene (Lacomme and Roby, 1996). No SOTs are found in the moss *Physcomitrella patens* or green alga *Chlamydomonas reinhardtii* revealing a late evolutionary origin of SOT.

Not all sulfated metabolites, however, are synthesized by SOTs, the most notable exceptions are the phytosulfokines and other sulfated peptides. These signaling compounds are modified at tyrosines, the reaction being catalyzed by tyrosylprotein sulfotransferase (TPST), which only recently has been discovered in plants (Komori et al., 2009). TPST is significantly different from SOTs: (1) it is localized in Golgi while SOTs are cytosolic, (2) it is unrelated to mammalian counterparts while SOTs structure is conserved, and (3) it is encoded by a single-copy gene in *Arabidopsis* in contrast to the SOT gene family (Klein and Papenbrock, 2004; Komori et al., 2009). Consequently, no obvious phenotypes can be observed in mutants of individual SOTs (own unpublished results), whereas *tpst* mutants are severely affected in growth (Komori et al., 2009). The common feature of tyrosine SOT and SOTs is the dependence on PAPS, which is synthesized by APS kinase.

## APS KINASE

Adenosine 5′-phosphosulfate kinase catalyzes the phosphorylation of APS to form PAPS. This enzyme is an essential component of primary sulfate assimilation of yeast, fungi, and some bacteria (Marzluf, 1997), which require this second activation of sulfate to enable its reduction by a PAPS reductase (Kopriva and Koprivova, 2004). While the cDNA for APS kinase were reported at the same time as those of other genes of sulfate assimilation (Arz et al., 1994; Jain and Leustek, 1994), the enzyme and its regulation was studied much less frequently compared to, e.g., ATP sulfurylase or APS reductase. A high affinity for APS (ca. 1–10 μM) was reported for APS kinase from *Arabidopsis* alongside a strong substrate inhibition (Lee and Leustek, 1998; Lillig et al., 2001). Only after the *Arabidopsis* genome sequence became available, the full family of four genes encoding APS kinase has been identified. All four genes encode functional enzymes with similarly high affinity for APS (Mugford et al., 2009). Three isoforms are localized in plastids and one, APK3, is cytosolic. *APK1* and *APK2* transcript levels in leaves are higher than those of *APK3* and APK4 (Mugford et al., 2009).

To find the biological functions of individual APS kinase isoforms, Mugford et al. (2009) systematically analyzed T-DNA lines disrupting the corresponding genes and their combinations. Unsurprisingly, disruptions of single genes had no effects on plant growth or contents of a major class of sulfated metabolites, glucosinolates. Among all six combinations of double mutants, only the *apk1 apk2* combination resulted in smaller plants in which glucosinolates reached ca. 15% of wild type levels (Mugford et al., 2009). Further crossing revealed that APS kinase isoforms, APK1, APK3, and APK4 alone are capable to sustain plant growth, albeit with great difference in performance (Mugford et al., 2010). Plants possessing APK1 as the only isoform of APS kinase were undistinguishable from wild type plants showing that this isoform contributes most to total enzyme activity. Plants possessing APK3 or APK4 only were affected in growth to a greater degree than *apk1 apk2* plants but were still capable to finish their life cycle and produce viable seeds. On the other hand, mutants with APK2 as the only active APS kinase isoform were not viable, most probably due to the lack of APK2 expression in pollen (Mugford et al., 2010). The analysis of APS kinase mutants thus revealed that this enzyme is essential for plants. It also showed that both plastidic and cytosolic PAPS production is capable to sustain growth, so that efficient transport of PAPS between these compartments has to be postulated. PAPS transporters located in Golgi apparatus have been identified in *Drosophila* (Lüders et al., 2003), and in mammals (Mandon et al., 1994), however, no PAPS transporter has been demonstrated in plants so far.

The differences in cellular localization and expression strength are not the only differences between the APS kinase isoforms. While the kinetic parameters of the four isoforms are almost identical (Mugford et al., 2009), their tissue-specific expression varies significantly. Apart from the lack of *APK2* transcript in pollen, where the other three isoforms are highly expressed, of particular interest is the high and very specific expression of *APK1* and *APK2* in funiculus and *APK3* and *APK4* in seed radical (Mugford et al., 2009). Analysis of available microarray data in eFP browser confirmed low expression levels of APS kinase isoforms and predominant expression of *APK2* in vegetative tissues (Winter et al., 2007). It also revealed that *APK1* and *APK2* are induced by methyl jasmonate, which agrees with up-regulation of these two genes by wounding (S. G. Mugford, unpublished). Interestingly, *APK1* transcript levels increased in imbibed seeds compared to dry controls, whereas *APK3* and *APK4* were down-regulated by this treatment (Nakabayashi et al., 2005). Thus, the differential tissue-specific expression of APS kinase isoforms together with the different growth characteristics of triple mutants indicates strongly that each isoform has a specific role in plant sulfur metabolism. However, to assess how APS kinase affects the general sulfur metabolism, it is necessary to consider the overall control of the flux through sulfate assimilation.

## APS REDUCTASE AS A KEY CONTROL STEP OF SULFUR METABOLISM

Adenosine 5′-phosphosulfate reductase has been studied extensively as the key enzyme controlling flux through reductive sulfate assimilation. The enzyme and the corresponding genes are highly regulated according to demand for reduced sulfur and sulfate availability. In particular, the enzyme is feedback inhibited by reduced sulfur compounds such as cysteine and glutathione (Vauclare et al., 2002). Since in the experiments with *Arabidopsis* root cultures this inhibition was specific to APS reductase and other enzymes of the pathway were not affected, the contribution of individual enzymes to total control of flux could be calculated. When internal sulfate was considered as the beginning of the pathway, APS reductase was responsible for 91% of control of the flux, when sulfate transport was also taken into account, the control was equally shared between transport and the enzyme (Vauclare et al., 2002). Using flux measurements with different transgenic poplars, APS reductase was again shown to possess a high control over the pathway, however not as strong as in case of *Arabidopsis*, contribution of other components of the pathway was clearly detectable (Scheerer et al., 2010).

**FIGURE 2:**
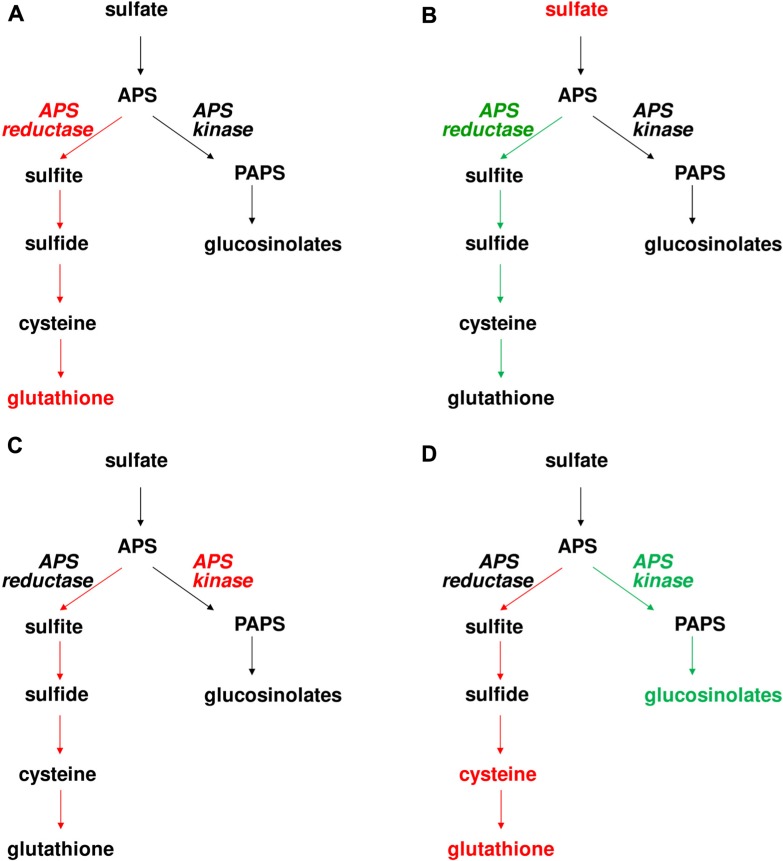
**Effects of manipulation of APS reductase and APS kinase on levels of S-containing compounds and flux through the pathway in *Arabidopsis***. Red and green colors symbolize increased and decreased enzyme activity, metabolite concentration, or flux through the pathway. The following genotypes were analyzed **(A)** *PpAPR-B* expressed in plastids, **(B)** *apr2*, **(C)** *EcAPK* targeted to plastids, and **(D)** *apk1 apk2.*

The data from control flux analysis showing importance of APS reductase in control of sulfate assimilation were corroborated by a very different experimental approach. To understand the control of sulfate accumulation in plants, analysis of Bay-0 × Shahdara recombinant inbred lines identified a major QTL on chromosome 1 (Loudet et al., 2007). The QTL was cloned as a gene encoding the APR2 isoform of APS reductase. In Shahdara, a single-nucleotide polymorphism results in exchange of an alanine in the proximity of active center into glutamate, leading to a strongly diminished affinity of the enzyme for the reductant and thus highly reduced reaction velocity. Since APR2 is the major isoform of the enzyme contributing about 75% of total leaf APS reductase activity, this polymorphism results in a very low APS reductase activity in Shahdara leaves and as a consequence, accumulation of sulfate (Loudet et al., 2007). This result was confirmed by analysis of *Arabidopsis* T-DNA line in which *APR2* gene was disrupted, as also this *apr2* mutant accumulated sulfate. Surprisingly, neither in Shahdara, nor in Col-0, the disruption of *APR2* and thus strongly reduced APS reductase activity affected the levels of reduced sulfur compounds, cysteine and glutathione (Loudet et al., 2007). This lack of effect, however, may be dependent on growth conditions, as the same *apr2* T-DNA line was reported to possess slightly but significantly lower glutathione level when grown under different conditions (Grant et al., 2011). Disruption of *APR2* may not affect glutathione levels, but the rate of its synthesis. Indeed, the flux through sulfate assimilation was diminished in the *apr2* mutants (**Figure 2**) (Mugford et al., 2011). It seems, therefore, that *Arabidopsis* is capable of maintaining stable glutathione levels by adjusting its turnover according to synthesis rate.

 While low APS reductase activity affects upstream metabolites (sulfate), it has only marginal effect on the reduced sulfur compounds. On the other hand, overexpression of APS reductase has significant adverse consequences for the plants. Expression of bacterial APS reductase in *Arabidopsis* and maize resulted in a strong accumulation of thiols, but also reduced inorganic sulfur compounds sulfite and thiosulfate (Tsakraklides et al., 2002). In maize, this metabolic unbalance led to leaf necrosis (Martin et al., 2005). Clearly, increased capacity for APS reduction results in increased flux of sulfur through reductive assimilation, but the accumulation of inorganic sulfur compounds shows that under these conditions the assimilation is limited by the availability of carbon acceptors of reduced sulfur. Indeed, feeding OAS to the APS reductase overexpressing *Arabidopsis* resulted in much higher accumulation of thiols (Tsakraklides et al., 2002). However, when the increase of APS reductase is only moderate, the effects on plant sulfur metabolism are much milder. This was shown with poplars overexpressing APS reductase from *Lemna minor*, which despite an increase in activity did not increase flux through the pathway. On the other hand, expression of APR-B form of APS reductase from *P. patens*, which does not possess iron sulfur cluster and is thus less catalytically efficient (Kopriva et al., 2007), has only marginal effect on the total enzyme activity but even this small rise is sufficient to enhance the flux through the pathway and even increase metabolite accumulation (**Figure 2**) (Mugford et al., 2011). This suggests that the increase in APS reductase activity by APR-B is uncoupled from the usual regulatory network and able to produce enough product surplus that the thiols accumulate. The effects of both reduced and increased APS reductase activities on flux through primary sulfate assimilation thus confirm the important role this enzyme has in the control of the pathway.

## APS KINASE AND SULFUR METABOLISM

As discussed previously, plants possess a range of sulfated compounds produced by secondary metabolism. Particularly in *Arabidopsis* and other Brassicaceae, the glucosinolates contribute significantly to sulfur pools in the plant and thus it can be expected that manipulation of APS kinase will have a general effect on sulfur metabolism. Indeed, the *Arabidopsis* *apk1 apk2* mutants with low glucosinolate levels showed remarkable increase in cysteine and glutathione (Mugford et al., 2009). Apparently, the block in the PAPS branch of sulfate assimilation caused a redirection of sulfur flow into the primary reductive pathway. Indeed, when the flux was quantified using incorporation of [^35^S]sulfate, the labeling of reduced sulfur compounds was higher in the mutant than in wild type (**Figure 2**) (Mugford et al., 2011). Surprisingly, the increased flux cannot be attributed to changes in APS reductase activity as this enzyme was not affected in the mutant. On the other hand, ATP sulfurylase activity was about twofold higher in *apk1 apk2* than in Col-0 (Mugford et al., 2009). This was in agreement with increased transcript accumulation of *ATPS1* and *ATPS3* isoforms of ATP sulfurylase in the mutant. Also sulfate uptake was increased in the mutants, which resulted in accumulation of sulfate (Mugford et al., 2009, 2011).

The reduction in APS kinase activity in *apk1 apk2* had consequences for other parts of sulfur metabolism as well. Microarray and qPCR analysis revealed that genes of the glucosinolate biosynthesis were highly and coordinately induced in the mutant (Mugford et al., 2009). Correspondingly, the mutants accumulated high levels of the desulfo-glucosinolate precursors. Similarly, the *apk1 apk2* plants possessed less sulfo-jasmonate and increased levels of the hydroxyl-jasmonate precursors. The genes encoding precursors of the sulfate peptides phytosulfokines and PSY1 were also more highly expressed in the mutants than in wild type (Mugford et al., 2009). The diminished availability of PAPS in the mutants thus affects all classes of sulfated products and leads to accumulation of the precursors.

As reduction in APS kinase affects both primary and secondary sulfur metabolism, what are the effects of increased PAPS production? While the levels of glucosinolates were not affected in plants overexpressing bacterial APS kinase, the manipulation of the enzyme level did not remain without consequences. Irrespective of localization of the additional APS kinase in cytosol or plastids, APS reductase activity was induced and consequently the flux through primary sulfate assimilation was increased (Mugford et al., 2011). Interestingly, the increase in sulfate reduction rate did not result in alteration of thiol accumulation (**Figure 2**). As with the *apr2* mutant, it seems that the levels of glutathione and cysteine are highly regulated within a narrow range by adjusting the synthesis and turnover rates. The increase in flux through primary sulfate assimilation and not the secondary branch in the APS kinase overexpressing plants seems rather counterintuitive. The most probable explanation is that increased APS kinase activity reduced the accumulation of APS and thus increased efficiency of ATP sulfurylase which is notorious for its low rate of forward (APS synthesis) reaction. The higher APS production then probably caused induction of APS reductase as the demand for the enzyme was higher and, consequently, the flux increased (Mugford et al., 2011).

## FURTHER LINKS BETWEEN PRIMARY AND SECONDARY SULFATE ASSIMILATION

An exciting new link between the two branches of sulfate assimilation has been uncovered very recently. The microarray analysis of *apk1 apk2* mutants indicated that a *FIERY1* gene, encoding a 2′(3′),5′-diphosphoadenosine (PAP) phosphatase, may be part of the glucosinolate synthesis network, because PAP is the product of SOT reactions (**Figure 1**) and the transcript for *FIERY1* was induced in *apk1 apk2* as the transcripts of other glucosinolate biosynthetic genes. Indeed, in leaves of a *fou8* allele of *fiery1 *(*fry1*) mutant (Rodríguez et al., 2010) glucosinolate content was reduced and desulfo-glucosinolates accumulated (Lee et al., 2012). Apparently, the PAP produced during glucosinolate synthesis accumulated in the *fou8* mutant and inhibited either the transport of PAPS between chloroplasts and cytosol or directly the SOT activity resulting in accumulation of the desulfo-precursors similar to *apk1 apk2*. However, the extent of reduction of glucosinolate levels and accumulation of desulfo-glucosinolates in *fou8* was much milder than in *apk1 apk2* mutants and the expression of genes of the glucosinolate synthesis network was only mildly affected (Lee et al., 2012). Analysis of *apk3 fou8* double mutants, in which the only cytosolic APS kinase was disrupted, revealed that it is not the PAPS transport causing the low glucosinolate phenotype, as their levels were identical to *fou8* plants. Thus, it seems that the accumulation of PAP in the *fou8* mutant inhibits SOTs and leads to reduced efficiency of desulfo-glucosinolate sulfation (Lee et al., 2012).

*FIERY1* is a rather enigmatic gene; it has been identified in numerous genetic screens for different phenotypes, such as screens for plant genes increasing Li^+^ tolerance of yeast (Quintero et al., 1996), affecting abscisic acid and stress signaling (Xiong et al., 2001), cold signaling (Xiong et al., 2004), RNA silencing suppressors (Gy et al., 2007), elevated expression of ascorbate peroxidase 2 (Wilson et al., 2009), venation patterning (Robles et al., 2010), deregulation of fatty acid oxygenation rate (Rodríguez et al., 2010), and for mutations affecting expression of a phosphate transporter (Hirsch et al., 2011). In *fry1* mutants PAP accumulates, causing large alterations in gene expression and inhibition of exoribonucleases (Estavillo et al., 2011). PAP levels are also highly induced by drought or high light stress acting as retrograde stress signals, from chloroplast to the nucleus to induce expression of stress-responsive genes. Indeed, *fry1* mutants are resistant to drought stress (Estavillo et al., 2011). The analysis of sulfur metabolism in *fou8* allele of *fry1* added another phenotype to the list, reduced accumulation of sulfate. The expression pattern of genes of primary sulfate assimilation in *fou8* is similar to that in plants under sulfate starvation: increased mRNA levels for APS reductase and reduced levels of *ATPS4* isoform of ATP sulfurylase (Lee et al., 2012). However, the expression pattern was not caused by disturbance in signaling, as the foliar sulfate and glutathione levels were significantly lower in *fou8* than in wild type. The mechanism by which sulfate levels are affected in *fou8* is not known, as the sulfate uptake rate to the roots is not affected (Lee et al., 2012). The strong correlation of sulfate starvation-like gene expression and low sulfate accumulation, however, indicate that the signal for induction of sulfate starvation response is reduction in internal rather than external sulfate content (Lee et al., 2012). Thus *FIERY1* or rather its substrate PAP accumulating in the *fou8* mutant represents another link between primary and secondary sulfate assimilation.

## REGULATORY MECHANISMS

The accumulation of desulfo-glucosinolates and coordinated induction of glucosinolate synthesis genes in the *apk1 apk2* mutant revealed that glucosinolate accumulation is under control of a feedback regulatory loop. The trigger for the regulation can be either the desulfo-glucosinolates, which are almost undetectable in wild type plants, or a decrease in glucosinolate(s) levels below a certain threshold. Since in the desulfo-glucosinolate accumulating *fou8* mutant the glucosinolate synthesis genes are not affected to the same degree as in *apk1 apk2*, it seems that *Arabidopsis* plants possess a mechanism reacting to low levels of one or more glucosinolates as a signal for induction of their synthesis. It is possible to speculate that binding of a certain glucosinolate, or its degradation product, to a transcription factor might prevent its binding to DNA. When the levels of such a signaling metabolite(s) are low, free transcription factors might be able to activate the gene expression of the glucosinolate synthesis network. An alternative signaling molecules might be PAP, which accumulates in nucleus during stress (Estavillo et al., 2011) or APS, which in bacteria binds to Cbl regulator and prevents Cbl-dependent transcription of genes for utilization of organic sulfur compounds (Bykowski et al., 2002). While the signal and its mechanism of action are unknown so far, transcription factors controlling the network are well established.

Glucosinolate synthesis is under control of two families of R2R3-MYB transcription factors (Gigolashvili et al., 2007a,b, 2008; Hirai et al., 2007; Sønderby, 2007). The first clade (MYB28, MYB76, and MYB29) is specifically involved in the control of synthesis of aliphatic glucosinolates (Gigolashvili et al., 2007b, 2008; Hirai et al., 2007; Sønderby, 2007) while MYB51, MYB122, and MYB34 regulate synthesis of indolic glucosinolates (Celenza et al., 2005; Gigolashvili et al., 2007a; Malitsky et al., 2008). Transcript levels of all these MYB factors are elevated in *apk1 apk2*, and the coordinated accumulation of mRNAs for the glucosinolate biosynthesis genes is in agreement with experiments in which these factors were overexpressed (Gigolashvili et al., 2007a,b, 2008; Mugford et al., 2009).

The MYB factors, however, function beyond regulation of genes of the core glucosinolate synthesis. The expression analysis of *apk1 apk2* plants revealed increased transcript levels of *ATPS1* and *ATPS3*. This prompted investigation of the ability of the MYB factors to control expression of genes of primary sulfate assimilation using *in vitro* transactivation assays (Berger et al., 2007). Given the dependence of glucosinolate synthesis on PAPS it is not surprising that *ATPS1*, *ATPS3*, *APK1*, and *APK2* are directly regulated by these transcription factors (Yatusevich et al., 2010). This has been confirmed by expression analysis of plants overexpressing these MYB factors. While both groups of the MYB factors controlled *APK1* and *APK2* to the same extent, the factors associated with aliphatic GSs, MYB28, MYB76, and MYB29, induced a stronger reaction with *ATPS1* than with *ATPS3*, while the opposite was true for all three indolic glucosinolate transcription factors (Yatusevich et al., 2010). Unexpectedly, the MYB factors appeared to regulate also genes of the reductive part of sulfate assimilation, APR and sulfite reductase. The regulation of APR by the MYB factors, however, is more complex. While *ATPS1* and *ATPS3* transcripts were induced in plants overexpressing the MYB factors and not affected in corresponding knock-out mutants, mRNA levels for APR were induced also in mutants in the MYB factors of the indolic glucosinolate group. It is possible that reduction of indolic glucosinolates in these mutants triggers synthesis of alternative defense compounds requiring reduced sulfur. Both disruption and overexpression of MYB28 and MYB51 had significant consequences for primary sulfate assimilation, including accumulation of glutathione and increased flux through the pathway (Yatusevich et al., 2010). Indeed, microarray analysis of plants overexpressing MYB28, MYB29, and MYB76 showed a similar regulation of genes of primary sulfate assimilation (Sønderby, 2007). It is thus clear that primary and secondary sulfate assimilation are interconnected and coordinated by these six MYB transcription factors.

Another factor regulating both primary and secondary sulfate assimilation is SULFUR LIMITATION 1 (SLIM1). SLIM1 has been identified in a screen for mutants in response to sulfate deficiency (Maruyama-Nakashita et al., 2006). The *slim1* mutants are not able to induce expression of high affinity sulfate transporter *SULTR1;2* and consequently sulfate uptake in sulfur limiting condition. Regulation of many but not all genes responsive to sulfate deficiency was altered in the mutants (Maruyama-Nakashita et al., 2006). Most notably, the up-regulation of APR was not altered in *slim1* plants. The flux through primary assimilation, however, was still affected by lack of SLIM1, probably due to regulation of ATP sulfurylase mediated through microRNA miR395, which is induced by sulfate deficiency in SLIM1-dependent manner (Kawashima et al., 2011). Apart from very strong effects on regulation of sulfate uptake and assimilation, SLIM1 also affects the secondary sulfate metabolism. One of the major responses to sulfur limitation is reduction in glucosinolate synthesis due to strong down-regulation of transcript levels of corresponding biosynthetic genes. In *slim1* mutants, the reduction in these transcripts is much less pronounced (Maruyama-Nakashita et al., 2006). Thus, SLIM1 controls the response of both branches of sulfate assimilation to sulfate limitation, however, the hierarchy of SLIM1 and the MYB factors involved in control glucosinolate synthesis still has to be established. SLIM1 is a member of *ETHYLENE-INSENSITIVE3-LIKE* family of transcription factors (Maruyama-Nakashita et al., 2006). Importantly, SLIM1 and its tobacco ortholog NtEIL2 activate promoters containing a UPE-box, which is present in eight *Arabidopsis* genes regulated by sulfate starvation (Wawrzyńska et al., 2010). While this finding is important in confirming the role of SLIM1 as a transcriptional activator, it does not explain the function of SLIM1 in control of sulfate starvation response, as among the genes containing the UPE-box are *APR1* and *APR3* that are not regulated by SLIM1 and on the other hand, the box is not present in promoter of *SULTR1;2*, which was used to in the genetic screen leading to SLIM1 discovery (Maruyama-Nakashita et al., 2006; Wawrzyńska et al., 2010). Therefore, despite some progress in characterization of SLIM1 and other proteins involved in regulation of sulfate starvation response, such as UP9 (Lewandowska et al., 2010), our understanding of mechanisms of response to sulfate starvation, including the function of SLIM1, is still largely incomplete.

However, there appears to be an additional mechanism regulating the partitioning of sulfur beyond the transcriptional regulation. It has been long known that APS reductase is redox regulated; the enzyme is activated during oxidative stress (Bick et al., 2001) and inactivated by incubation with excess reductant (Kopriva and Koprivova, 2004). Very recently it has been shown that also APS kinase is susceptible to redox regulation (Ravilious et al., 2012). Resolving the protein structure of APK1 revealed that the enzyme possesses a disulfide bond between conserved cysteines. Reduction of the disulfide either chemically or via site-directed mutagenesis resulted in increased activity and lower substrate inhibition. It seems therefore, that changes in redox environment in the plastids may change the flow of sulfur to more reduced products when oxidized and to more secondary assimilation when sufficient reduction equivalents are available (Ravilious et al., 2012). Such a mechanism ensures that abiotic stress, connected with production of reactive oxygen species and more oxidizing conditions, stimulates sulfate reduction through activation of APR and inhibition of APS kinase.

In conclusion, it is evident that both APS reductase and APS kinase are capable of regulating the flux through sulfate assimilation and that it is the coordination of these two activities that is responsible for control of partitioning of sulfur between primary and secondary metabolism. The coordination is achieved on several levels, transcriptional regulation through a common set of MYB transcription factors and post-translational vie redox sensitive cysteine bonds in the enzymes. While the progress in understanding of the control of this partitioning has been admirable, we are still far from exploitation of these finding in praxis to manipulate the synthesis of sulfur-containing compounds in plants by genetic engineering. This will certainly be a target of further studies, as demonstrated, e.g., by the ability to engineer glucosinolate synthesis to tobacco (Geu-Flores et al., 2009). The time for designer crops with manipulated contents of specific sulfur-containing metabolites is coming!

## Conflict of Interest Statement

The authors declare that the research was conducted in the absence of any commercial or financial relationships that could be construed as a potential conflict of interest.

## References

[B1] Amano Y. Tsubouchi H. Shinohara H. Ogawa M. Matsubayashi Y. (2007). Tyrosine-sulfated glycopeptide involved in cellular proliferation and expansion in *Arabidopsis*. *Proc. Natl. Acad. Sci. U.S.A.* 104 18333–18338 17989228 10.1073/pnas.0706403104 PMC2084343

[B2] Arz H. E. Gisselmann G. Schiffmann S. Schwenn J. D. (1994). A cDNA for adenylyl sulphate (APS)-kinase from *Arabidopsis thaliana*. *Biochim. Biophys. Acta* 1218 447–452 8049272 10.1016/0167-4781(94)90203-8

[B3] Baek D. Pathange P. Chung J. S. Jiang J. Gao L. Oikawa A. Hirai M. Y. Saito K. Pare P. W. Shi H. (2010). A stress-inducible sulphotransferase sulphonates salicylic acid and confers pathogen resistance in *Arabidopsis*. *Plant Cell Environ.* 33 1383–1392 20374532 10.1111/j.1365-3040.2010.02156.x

[B4] Barron D. Varin L. Ibrahim R. K. Harborne J. B. Williams C. A. (1988). Sulphated flavonoids – an update. *Phytochemistry* 27 2375–2395

[B5] Berger B. Stracke R. Yatusevich R. Weisshaar B. Flügge U. I. Gigolashvili T. (2007). A simplified method for the analysis of transcription factor-promoter interactions that allows high-throughput data generation. *Plant J.* 50 911–916 17425717 10.1111/j.1365-313X.2007.03088.x

[B6] Bick J. A. Setterdahl A. T. Knaff D. B. Chen Y. Pitcher L. H. Zilinskas B. A. Leustek T. (2001). Regulation of the plant-type 5′-adenylyl sulfate reductase by oxidative stress. *Biochemistry* 40 9040–9048 11467967 10.1021/bi010518v

[B7] Buchner P. Takahashi H. Hawkesford M. J. (2004). Plant sulphate transporters: co-ordination of uptake, intracellular and long-distance transport. *J. Exp. Bot.* 55 1765–1773 15258169 10.1093/jxb/erh206

[B8] Bykowski T. van der Ploeg J. R. Iwanicka-Nowicka R. Hryniewicz M. M. (2002). The switch from inorganic to organic sulphur assimilation in *Escherichia coli*: adenosine 5′-phosphosulphate (APS) as a signalling molecule for sulphate excess. *Mol. Microbiol.* 43 1347–1358 11918818 10.1046/j.1365-2958.2002.02846.x

[B9] Celenza J. L. Quiel J. A. Smolen G. A. Merrikh H. Silvestro A. R. Normanly J. Bender J. (2005). The *Arabidopsis* ATR1 Myb transcription factor controls indolic glucosinolate homeostasis. *Plant Physiol.* 137 253–262 15579661 10.1104/pp.104.054395 PMC548856

[B10] Coughtrie M. W. H. Sharp S. Maxwell K. Innes N. P. (1998). Biology and function of the reversible sulfation pathway catalysed by human sulfotransferases and sulfatases. *Chem. Biol. Interact.* 109 3–27 9566730 10.1016/s0009-2797(97)00117-8

[B11] Estavillo G. M. Crisp P. A. Pornsiriwong W. Wirtz M. Collinge D. Carrie C. Giraud E. Whelan J. David P. Javot H. Brearley C. Hell R. Marin E. Pogson B. J. (2011). Evidence for a SAL1-PAP chloroplast retrograde pathway that functions in drought and high light signaling in *Arabidopsis*. *Plant Cell* 23 3992–4012 22128124 10.1105/tpc.111.091033 PMC3246320

[B12] Fahey J. W. Zalcmann A. T. Talalay P. (2001). The chemical diversity and distribution of glucosinolates and isothiocyanates among plants. *Phytochemistry* 56 5–51 11198818 10.1016/s0031-9422(00)00316-2

[B13] Geu-Flores F. Nielsen M. T. Nafisi M. Møldrup M. E. Olsen C. E. Motawia M. S. Halkier B. A. (2009). Glucosinolate engineering identifies a gamma-glutamyl peptidase. *Nat. Chem. Biol.* 5 575–577 19483696 10.1038/nchembio.185

[B14] Gidda S. K. Miersch O. Levitin A. Schmidt J. Wasternack C. Varin L. (2003). Biochemical and molecular characterization of a hydroxyjasmonate sulfotransferase from *Arabidopsis thaliana*. *J. Biol. Chem.* 278 17895–17900 12637544 10.1074/jbc.M211943200

[B15] Gidda S. K. Varin L. (2006). Biochemical and molecular characterization of flavonoid 7-sulfotransferase from *Arabidopsis thaliana*. *Plant Physiol. Biochem.* 44 628–636 17095238 10.1016/j.plaphy.2006.10.004

[B16] Gigolashvili T. Berger B. Mock H. P. Müller C. Weisshaar B Flügge U. I. (2007a). The transcription factor HIG1/MYB51 regulates indolic glucosinolate biosynthesis in *Arabidopsis thaliana*. *Plant J.* 50 886–901 17461791 10.1111/j.1365-313X.2007.03099.x

[B17] Gigolashvili T. Yatusevich R. Berger B. Müller C Flügge U. I. (2007b). The R2R3-MYB transcription factor HAG1/MYB28 is a regulator of methionine-derived glucosinolate biosynthesis in *Arabidopsis thaliana*. *Plant J.* 51 247–261 17521412 10.1111/j.1365-313X.2007.03133.x

[B18] Gigolashvili T. Engqvist M. Yatusevich R. Müller C Flügge U. I. (2008). HAG2/MYB76 and HAG3/MYB29 exert a specific and coordinated control on the regulation of aliphatic glucosinolate biosynthesis in *Arabidopsis thaliana*. *New Phytol.* 177 627–642 18042203 10.1111/j.1469-8137.2007.02295.x

[B19] Grant K. Carey N. M. Mendoza M. Schulze J. Pilon M. Pilon-Smits E. A van Hoewyk D. (2011). Adenosine 5′-phosphosulfate reductase (APR2) mutation in *Arabidopsis* implicates glutathione deficiency in selenate toxicity. *Biochem. J.* 438 325–335 21585336 10.1042/BJ20110025

[B20] Gy I. Gasciolli V. Lauressergues D. Morel J. B. Gombert J. Proux F. Proux C. Vaucheret H. Mallory A. C. (2007). *Arabidopsis* FIERY1, XRN2, and XRN3 are endogenous RNA silencing suppressors. *Plant Cell* 19 3451–3461 17993620 10.1105/tpc.107.055319 PMC2174888

[B21] Haas F. H. Heeg C. Queiroz R. Bauer A. Wirtz M. Hell R. (2008). Mitochondrial serine acetyltransferase functions as a pacemaker of cysteine synthesis in plant cells. *Plant Physiol.* 148 1055–1067 18753283 10.1104/pp.108.125237 PMC2556817

[B22] Halkier B. A. Gershenzon J. (2006). Biology and biochemistry of glucosinolates. *Annu. Rev. Plant Biol.* 57 303–333 16669764 10.1146/annurev.arplant.57.032905.105228

[B23] Hirai M. Y. Klein M. Fujikawa Y. Yano M. Goodenowe D. B. Yamazaki Y. Kanaya S. Nakamura Y. Kitayama M. Suzuki H. Sakurai N. Shibata D. Tokuhisa J. Reichelt M. Gershenzon J. Papenbrock J. Saito K. (2005). Elucidation of gene-to-gene and metabolite-to-gene networks in *Arabidopsis* by integration of metabolomics and transcriptomics. *J. Biol. Chem.* 280 25590–25595 15866872 10.1074/jbc.M502332200

[B24] Hirai M. Y. Sugiyama K. Sawada Y. Tohge T. Obayashi T. Suzuki A. Araki R. Sakurai N. suzuki H. Aoki K. Goda H. Nishizawa O. I. Shibata D. Saito K. (2007). Omics-based identification of *Arabidopsis* Myb transcription factors regulating aliphatic glucosinolate biosynthesis. *Proc. Natl. Acad. Sci. U.S.A.* 104 6478–6483 17420480 10.1073/pnas.0611629104 PMC1849962

[B25] Hirsch J. Misson J. Crisp P. A. David P. Bayle V. Estavillo G. M. Javot H. Chiarenza S. Mallory A. C. Maizel A. Declerck M. Pogson B. J. Vaucheret H. Crespi M. Desnos T. Thibaud M. C. Nussaume L. Marin E. (2011). A novel *fry1* allele reveals the existence of a mutant phenotype unrelated to 5′ → 3′ exoribonuclease (XRN) activities in *Arabidopsis thaliana* roots. *PLoS ONE* 6 e16724 10.1371/journal.pone.0016724 PMC3033419 21304819

[B26] Jain A. Leustek T. (1994). A cDNA clone for 5′-adenylylphosphosulfate kinase from *Arabidopsis thaliana*. *Plant Physiol.* 105 771–772 8066145 10.1104/pp.105.2.771 PMC159428

[B27] Kawashima C. G. Matthewman C. A. Huang S. Lee B.-R. Yoshimoto N. Koprivova A. Rubio-Somoza I. Todesco M. Rathjen T. Saito K. Takahashi H. Dalmay T. Kopriva S. (2011). Interplay of SLIM1 and miR395 in the regulation of sulfate assimilation in *Arabidopsis*. *Plant J.* 66 863–876 21401744 10.1111/j.1365-313X.2011.04547.x

[B28] Khan M. S. Haas F. H. Samami A. A. Gholami A. M. Bauer A. Fellenberg K. Reichelt M. Hänsch R. Mendel R. R. Meyer A. J. Wirtz M. Hell R. (2010). Sulfite reductase defines a newly discovered bottleneck for assimilatory sulfate reduction and is essential for growth and development in *Arabidopsis thaliana*. *Plant Cell* 22 1216–1231 20424176 10.1105/tpc.110.074088 PMC2879758

[B29] Klein M. Papenbrock J. (2004). The multi-protein family of *Arabidopsis* sulfotransferases and their relatives in other plant species. *J. Exp. Bot.* 55 1809–1820 15234990 10.1093/jxb/erh183

[B30] Klein M. Reichelt M. Gershenzon J. Papenbrock J. (2006). The three desulfoglucosinolate sulfotransferase proteins in *Arabidopsis* have different substrate specificities and are differentially expressed. *FEBS J.* 273 122–136 16367753 10.1111/j.1742-4658.2005.05048.x

[B31] Komori R. Amano Y. Ogawa-Ohnishi M. Matsubayashi Y. (2009). Identification of tyrosylprotein sulfotransferase in *Arabidopsis*. *Proc. Natl. Acad. Sci. U.S.A.* 106 15067–15072 19666544 10.1073/pnas.0902801106 PMC2736448

[B32] Kopriva S. Fritzemeier K. Wiedemann G. Reski R. (2007). The putative moss 3′-phosphoadenosine-5′-phosphosulfate reductase is a novel form of adenosine-5′-phosphosulfate reductase without an iron-sulfur cluster. *J. Biol. Chem.* 282 22930–22938 17519237 10.1074/jbc.M702522200

[B33] Kopriva S. Koprivova A. (2004). Plant adenosine 5′-phosphosulphate reductase: the past, the present, and the future. *J. Exp. Bot.* 55 1775–1783 15208336 10.1093/jxb/erh185

[B34] Kopriva S. Mugford S. G. Matthewman C. A. Koprivova A. (2009). Plant sulfate assimilation genes: redundancy vs. specialization. *Plant Cell Rep.* 28 1769–1780 10.1007/s00299-009-0793-0 19876632

[B35] Lacomme C. Roby D. (1996). Molecular cloning of a sulfotransferase in *Arabidopsis thaliana* and regulation during development and in response to infection with pathogenic bacteria. *Plant Mol. Biol.* 30 995–1008 8639757 10.1007/BF00020810

[B36] Lee B.-R. Huseby S. Koprivova A. Chételat A. Wirtz M. Mugford S. T. Navid E. Brearley C. Saha S. Mithen R. Hell R. Farmer E. E. Kopriva S. (2012). Effects of fou8/fry1 mutation on sulfur metabolism: is decreased internal sulfate the trigger of sulfate starvation response? *PLoS ONE* 7 e39425. 10.1371/journal.pone.0039425 PMC3377649 22724014

[B37] Lee S. Leustek T. (1998). APS kinase from *Arabidopsis thaliana*: genomic organization, expression, and kinetic analysis of the recombinant enzyme. *Biochem. Biophys. Res. Commun.* 247 171–175 9636674 10.1006/bbrc.1998.8751

[B38] Lewandowska M. Wawrzynska A. Moniuszko G. Lukomska J. Zientara K. Piecho M. Hodurek P. Zhukov I. Liszewska F. Nikiforova V. Sirko A. (2010). A contribution to identification of novel regulators of plant response to sulfur deficiency: characteristics of a tobacco gene UP9C, its protein product and the effects of UP9C silencing. *Mol. Plant* 3 347–360 20147370 10.1093/mp/ssq007 PMC2845781

[B39] Lillig C. H. Schiffmann S. Berndt C. Berken A. Tischka R. Schwenn J. D. (2001). Molecular and catalytic properties of *Arabidopsis thaliana* adenylyl sulfate (APS)-kinase. *Arch. Biochem. Biophys.* 392 303–310 11488606 10.1006/abbi.2001.2453

[B40] Loudet O. Saliba-Colombani V. Camilleri C. Calenge F. Gaudon V. Koprivova A. North K. A. Kopriva S. Daniel-Vedele F. (2007). Natural variation for sulfate content in *Arabidopsis thaliana* is highly controlled by APR2. *Nat. Genet.* 39 896–900 17589509 10.1038/ng2050

[B41] Lüders F. Segawa H. Stein D. Selva E. M. Perrimon N. Turco S. J Häcker U. (2003). Slalom encodes an adenosine 3′-phosphate 5′-phosphosulfate transporter essential for development in *Drosophila*. *EMBO J.* 22 3635–3644 12853478 10.1093/emboj/cdg345 PMC165615

[B42] Malitsky S. Blum E. Less H. Venger I. Elbaz M. Morin S. Eshed Y. Aharoni A. (2008). The transcript and metabolite networks affected by the two clades of *Arabidopsis* glucosinolate biosynthesis regulators. *Plant Physiol.* 148 2021–2049 18829985 10.1104/pp.108.124784 PMC2593676

[B43] Mandon E. C. Milla M. E. Kempner E. Hirschberg C. B. (1994). Purification of the Golgi adenosine 3′-phosphate 5′-phosphosulfate transporter, a homodimer within the membrane. *Proc. Natl. Acad. Sci. U.S.A.* 91 10707–10711 7938015 10.1073/pnas.91.22.10707 PMC45091

[B44] Marsolais F. Boyd J. Paredes Y. Schinas A. M. Garcia M. Elzein S. Varin L. (2007). Molecular and biochemical characterization of two brassinosteroid sulfotransferases from *Arabidopsis*, AtST4a (At2g14920) and AtST1 (At2g03760). *Planta* 225 1233–1244 17039368 10.1007/s00425-006-0413-y

[B45] Martin M. N. Tarczynski M. C. Shen B. Leustek T. (2005). The role of 5′-adenylylsulfate reductase in controlling sulfate reduction in plants. *Photosynth. Res.* 86 309–323 16328785 10.1007/s11120-005-9006-z

[B46] Maruyama-Nakashita A. Nakamura Y. Tohge T. Saito K. Takahashi H. (2006). *Arabidopsis* SLIM1 is a central transcriptional regulator of plant sulfur response and metabolism. *Plant Cell* 18 3235–3251 17114350 10.1105/tpc.106.046458 PMC1693955

[B47] Marzluf G. A. (1997). Molecular genetics of sulfur assimilation in filamentous fungi and yeast. *Annu. Rev. Microbiol.* 51 73–96 9343344 10.1146/annurev.micro.51.1.73

[B48] Matsubayashi Y. Sakagami Y. (1996). Phytosulfokine, sulfated peptides that induce the proliferation of single mesophyll cells of *Asparagus officinalis* L. *Proc. Natl. Acad. Sci. U.S.A.* 93 7623–7627 8755525 10.1073/pnas.93.15.7623 PMC38796

[B49] Matsuzaki Y. Ogawa-Ohnishi M. Mori A. Matsubayashi Y. (2010). Secreted peptide signals required for maintenance of root stem cell niche in *Arabidopsis*. *Science* 329 1065–1067 20798316 10.1126/science.1191132

[B50] Ménard R. Alban S. de Ruffray P. Jamois F. Franz G. Fritig B. Yvin J. C. Kauffmann S. (2004). Beta-1,3 glucan sulfate, but not beta-1,3 glucan, induces the salicylic acid signaling pathway in tobacco and *Arabidopsis*. *Plant Cell* 16 3020–3032 15494557 10.1105/tpc.104.024968 PMC527195

[B51] Mithen R. Faulkner K. Magrath R. Rose P. Williamson G. Marquez J. (2003). Development of isothiocyanate-enriched broccoli, and its enhanced ability to induce phase 2 detoxification enzymes in mammalian cells. *Theor. Appl. Genet.* 106 727–734 12596003 10.1007/s00122-002-1123-x

[B52] Mugford S. G. Lee B.-R. Koprivova A. Matthewman C. Kopriva S. (2011). Control of sulfur partitioning between primary and secondary metabolism. *Plant J.* 65 96–105 21175893 10.1111/j.1365-313X.2010.04410.x

[B53] Mugford S. G. Matthewman C. A. Hill L. Kopriva S. (2010). Adenosine 5′ phosphosulfate kinase is essential for *Arabidopsis* viability. *FEBS Lett.* 584 119–123 19903478 10.1016/j.febslet.2009.11.014

[B54] Mugford S. G. Yoshimoto N. Reichelt M. Wirtz M. Hill L. Mugford S. T. Nakazato Y. Noji M. Takahashi H. Kramell R. Gigolashvili T. Flügge U.-I. Wasternack C. Gershenzon J. Hell R. Saito K. Kopriva S. (2009). Disruption of adenosine-5′-phosphosulfate kinase in *Arabidopsis* reduces levels of sulfated secondary metabolites. *Plant Cell* 21 910–927 19304933 10.1105/tpc.109.065581 PMC2671714

[B55] Nakabayashi K. Okamoto M. Koshiba T. Kamiya Y. Nambara E. (2005). Genome-wide profiling of stored mRNA in *Arabidopsis thaliana* seed germination: epigenetic and genetic regulation of transcription in seed. *Plant J.* 41 697–709 15703057 10.1111/j.1365-313X.2005.02337.x

[B56] Patron N. J. Durnford D. G. Kopriva S. (2008). Sulfate assimilation in eukaryotes: fusions, relocations and lateral transfers. *BMC Evol. Biol.* 8 39 10.1186/1471-2148-8-39 PMC2275785 18248682

[B57] Piotrowski M. Schemenewitz A. Lopukhina A. Müller A. Janowitz T. Weiler E. W. Oecking C. (2004). Desulfoglucosinolate sulfotransferases from *Arabidopsis thaliana *catalyze the final step in the biosynthesis of the glucosinolate core structure. *J. Biol. Chem.* 279 50717–50725 15358770 10.1074/jbc.M407681200

[B58] Quintero F. J. Garciadeblás, B Rodríguez-Navarro A. (1996). The SAL1 gene of *Arabidopsis*, encoding an enzyme with 3′(2′),5′-bisphosphate nucleotidase and inositol polyphosphate 1-phosphatase activities, increases salt tolerance in yeast. *Plant Cell* 8 529–537 8721754 10.1105/tpc.8.3.529 PMC161118

[B59] Ravilious G. E. Nguyen A. Francois J. A. Jez J. M. (2012). Structural basis and evolution of redox regulation in plant adenosine-5′-phosphosulfate kinase. *Proc. Natl. Acad. Sci. U.S.A.* 109 309–314 22184237 10.1073/pnas.1115772108 PMC3252903

[B60] Robles P. Fleury D. Candela H. Cnops G. Alonso-Peral M. M. Anami S. Falcone A. Caldana C. Willmitzer L. Ponce M. R. Van Lijsebettens M. Micol J. L. (2010). The RON1/FRY1/SAL1 gene is required for leaf morphogenesis and venation patterning in *Arabidopsis*. *Plant Physiol.* 152 1357–1372 20044451 10.1104/pp.109.149369 PMC2832283

[B61] Rodríguez V. M. Chételat A. Majcherczyk P. Farmer E. E. (2010). Chloroplastic phosphoadenosine phosphosulfate metabolism regulates basal levels of the prohormone jasmonic acid in *Arabidopsis* leaves. *Plant Physiol.* 152 1335–1345 20053710 10.1104/pp.109.150474 PMC2832275

[B62] Scheerer U. Haensch R. Mendel R. R. Kopriva S. Rennenberg H. Herschbach C. (2010). Sulphur flux through the 1 sulphate assimilation pathway is differently controlled by adenosine 5′-phosphosulphate reductase under stress and in transgenic poplar plants overexpressing γ-ECS, SO or APR. *J. Exp. Bot.* 61 609–622 19923196 10.1093/jxb/erp327 PMC2803220

[B63] Sønderby I. E. Hansen B. G. Bjarnholt N. Ticconi C. Halkier B. A. Kliebenstein D. J. (2007). A systems biology approach identifies a R2R3 MYB gene subfamily with distinct and overlapping functions in regulation of aliphatic glucosinolates. *PLoS ONE* 2 e1322 10.1371/journal.pone.0001322 PMC2147653 18094747

[B64] Takahashi H. Kopriva S. Giordano M. Saito K. Hell R. (2011). Sulfur assimilation in photosynthetic organisms: molecular functions and regulations of transporters and assimilatory enzymes. *Annu. Rev. Plant Biol.* 62 157–184 21370978 10.1146/annurev-arplant-042110-103921

[B65] Truchet G. Roche P. Lerouge P. Vasse J. Camut S. de Billy F. Promé J.-C. Dénarié J. (1991). Sulphated lipo-oligosaccharide signals of *Rhizobium meliloti* elicit root nodule organogenesis in alfalfa. *Nature* 351 670–673

[B66] Tsakraklides G. Martin M. Chalam R. Tarczynski M. C. Schmidt A. Leustek T. (2002). Sulfate reduction is increased in transgenic *Arabidopsis thaliana* expressing 5′-adenylylsulfate reductase from *Pseudomonas aeruginosa*. *Plant J.* 32 879–889 12492831 10.1046/j.1365-313x.2002.01477.x

[B67] Varin L. Marsolais F. Richard M. Rouleau M. (1997). Biochemistry and molecular biology of plant sulfotransferases. *FASEB J.* 11 517–525 9212075 10.1096/fasebj.11.7.9212075

[B68] Vauclare P. Kopriva S. Fell D. Suter M. Sticher L. von Ballmoos O. Krähenbuhl U. Op den Camp R. Brunold C. (2002). Flux control of sulfate assimilation in *Arabidopsis thaliana*: adenosine 5′-phosphosulfate reductase is more susceptible to negative control by thiols than ATP sulfurylase. *Plant J.* 31 729–740 12220264 10.1046/j.1365-313x.2002.01391.x

[B69] Watanabe M. Mochida K. Kato T. Tabata S. Yoshimoto N. Noji M. Saito K. (2008). Comparative genomics and reverse genetics analysis reveal indispensable functions of the serine acetyltransferase gene family in *Arabidopsis*. *Plant Cell* 20 2484–2496 18776059 10.1105/tpc.108.060335 PMC2570737

[B70] Wawrzyńska A. Lewandowska M. Sirko A. (2010). *Nicotiana tabacum* EIL2 directly regulates expression of at least one tobacco gene induced by sulphur starvation. *J. Exp. Bot.* 61 889–900 20018902 10.1093/jxb/erp356

[B71] Wilson P. B. Estavillo G. M. Field K. J. Pornsiriwong W. Carroll A. J. Howell K. A. Woo N. S. Lake J. A. Smith S. M. Harvey Millar A. von Caemmerer S. Pogson B. J. (2009). The nucleotidase/phosphatase SAL1 is a negative regulator of drought tolerance in *Arabidopsis*. *Plant J.* 58 299–317 19170934 10.1111/j.1365-313X.2008.03780.x

[B72] Winter D. Vinegar B. Nahal H. Ammar R. Wilson G. V. Provart N. J. (2007). An “Electronic Fluorescent Pictograph” browser for exploring and analyzing large-scale biological data sets. *PLoS ONE* 2 e718 10.1371/journal.pone.0000718 PMC1934936 17684564

[B73] Xiong L. Lee B. H. Ishitani M. Lee H. Zhang C. Zhu J. K. (2001). *FIERY1* encoding an inositol polyphosphate 1-phosphatase is a negative regulator of abscisic acid and stress signaling in *Arabidopsis*. *Genes Dev.* 15 1971–1984 11485991 10.1101/gad.891901 PMC312749

[B74] Xiong L. Lee H. Huang R. Zhu J. K. (2004). A single amino acid substitution in the *Arabidopsis* FIERY1/HOS2 protein confers cold signaling specificity and lithium tolerance. *Plant J.* 40 536–545 15500469 10.1111/j.1365-313X.2004.02225.x

[B75] Yatusevich R. Mugford S. G. Matthewman C. Gigolashvili T. Frerigmann H. Delaney S. Koprivova A. Flügge U.-I. Kopriva S. (2010). Genes of primary sulfate assimilation are part of the glucosinolate biosynthetic network in *Arabidopsis thaliana*. *Plant J.* 62 1–11 20042022 10.1111/j.1365-313X.2009.04118.x

